# Human and mouse PD-L1: similar molecular structure, but different druggability profiles

**DOI:** 10.1016/j.isci.2020.101960

**Published:** 2020-12-24

**Authors:** Katarzyna Magiera-Mularz, Justyna Kocik, Bogdan Musielak, Jacek Plewka, Dominik Sala, Monika Machula, Przemyslaw Grudnik, Malgorzata Hajduk, Marcin Czepiel, Maciej Siedlar, Tad A. Holak, Lukasz Skalniak

**Affiliations:** 1 Department of Organic Chemistry, Faculty of Chemistry, Jagiellonian University, Gronostajowa 2, 30-387 Krakow, Poland; 2 Malopolska Center of Biotechnology Jagiellonian University, Gronostajowa 7, 30-387 Krakow, Poland; 3 Department of Clinical Immunology, Institute of Pediatrics, Jagiellonian University Medical College, Wielicka 265, 30-663 Krakow, Poland

**Keywords:** Therapeutics, Immunology

## Abstract

In the development of PD-L1-blocking therapeutics, it is essential to transfer initial *in vitro* findings into proper *in vivo* animal models. Classical immunocompetent mice are attractive due to high accessibility and low experimental costs. However, it is unknown whether inter-species differences in PD-L1 sequence and structure would allow for human-mouse cross applications. Here, we disclose the first structure of the mouse (*m*) PD-L1 and analyze its similarity to the human (*h*) PD-L1. We show that *m*PD-L1 interacts with *h*PD-1 and provides a negative signal toward activated Jurkat T cells. We also show major differences in druggability between the *h*PD-L1 and *m*PD-L1 using therapeutic antibodies, a macrocyclic peptide, and small molecules. Our study indicates that while the amino acid sequence is well conserved between the *h*PD-L1 and *m*PD-L1 and overall structures are almost identical, crucial differences determine the interaction with anti-PD-L1 agents, that cannot be easily predicted *in silico*.

## Highlights

• Mouse (*m*) PD-L1 interacts with human (*h*) PD-1 and inhibits human Jurkat T cells
• Small molecule and macrocyclic peptide inhibitors of *h*PD-L1 do not bind to *m*PD-L1
• Atezolizumab but not durvalumab binds and blocks mouse PD-L1

## Graphical abstract

**Figure:**
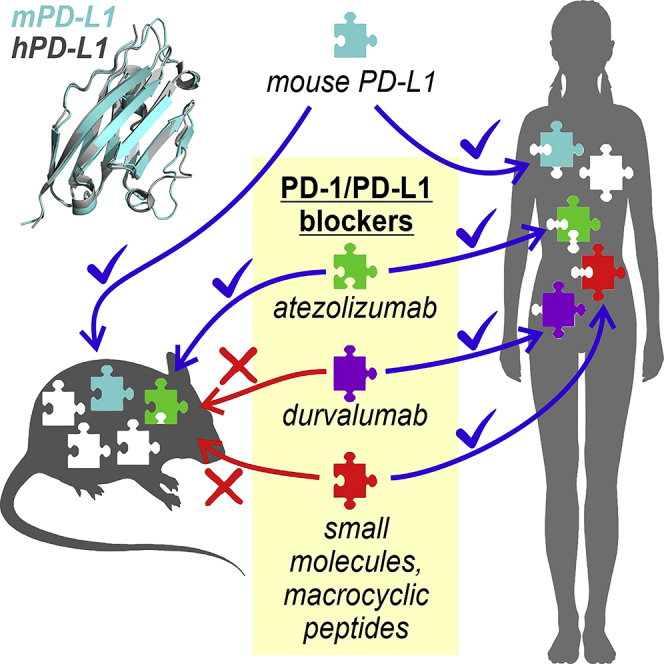

## Introduction

Tumor cells demonstrate tumor-specific antigens that facilitate the recognition by immune cells, provide the activation of the immune system, and enable the elimination of transformed cells. Immune checkpoint receptors play a crucial role in maintaining physiological T cell homeostasis, but also constitute a major factor in the escape of tumor cells from immune surveillance. The expression of immune checkpoint proteins allows for cancer progression by providing inhibitory circuits toward tumor-reactive T cells (Friedl and Wolf, 2003; Topalian et al., 2015; Zitvogel et al., 2006).

Programmed cell death receptor 1 (PD-1, CD279), together with its ligand, PD-L1 (CD274, B7-H1), constitutes one of the most important immune checkpoints as therapeutic targets (Azuma et al., 2008; Francisco et al., 2009; Zhang et al., 2009). Accordingly, over the past few years cancer immunotherapies based on therapeutic antibodies that block either the PD-1 receptor expressed on effector cells of the immune system or PD-L1 on tumor cells or in the tumor microenvironment, have revolutionized the approach to cancer treatment (Hoos, 2016) and provided a positive therapeutic outcome in a significant subset of patients (Chae et al., 2018; Herbst et al., 2014; Larkin et al., 2019). Until now, six monoclonal antibodies targeting PD-1/PD-L1 interaction have been approved by the US Food and Drug Administration (FDA) (Lee et al., 2019).

The clinical success of antibody-driven therapies evoked a great pursuit for the discovery of small-molecule- and peptide-based PD-1/PD-L1 blockers (Guzik et al., 2019; Shaabani et al., 2018; Yang and Hu, 2019), which would reduce the costs of therapies and possibly allow for *per os* drug delivery (Hansel et al., 2010). Transferring drug candidates through *in vivo* experiments with the use of appropriate animal models is a fundamental process that allows for the evaluation of the therapeutic index and progression to clinical trials. While in the classical experimental chemotherapy and targeted therapy approaches immunodeficient mice bearing human cell xenografts are largely acceptable models, immunotherapy-directed studies require the co-existence of the functional immune system and immune-compatible tumor implants. This can be achieved either by using syngeneic mouse models (fully mouse systems) or by introducing effortful humanized animals that provide the interaction between human target cells, or at least human molecular targets (De La Rochere et al., 2018; Zitvogel et al., 2016). Importantly, the choice of a proper model has to be made rationally following the analysis of the druggability of the molecular target of human and mouse origins. However, structural details on the mouse PD-L1 (*m*PD-L1), as well as *in vitro* methods for the prediction and verification of its applicability in particular immuno-oncology projects are still missing.

Here, for the first time, we deliver the structure of the PD-1-binding domain of *m*PD-L1. Next we show that alike its human counterpart, *m*PD-L1 is able to interact with the *hPD*-1 and provides a functional immune checkpoint for the activated human Jurkat T cells. Then, we check the druggability of the *h*/*h* and *h*/*m* PD-1/PD-L1 immune checkpoints with representative PD-L1-targeting small molecules and a macrocyclic peptide. We also demonstrate the use of a new cell-based assay that may guide the selection between the classical mouse models and humanized mouse models for pre-clinical testing of the activity of experimental PD-L1-targeting drugs.

## Results

### The overall structure of the mouse Apo-PD-L1

**Figure 1:**
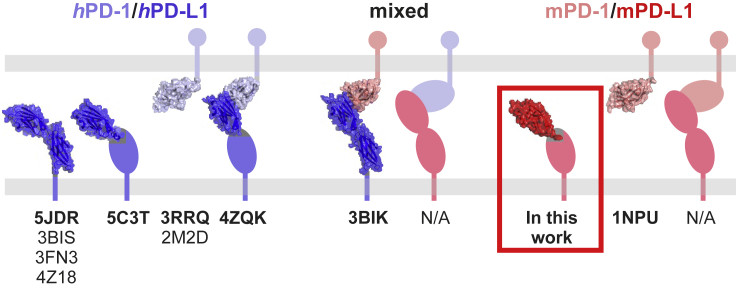
The available molecular structures that comprise either the human or mouse PD-1 or PD-L1 proteins alone or complexes thereof

Several molecular structures of human PD-1 and PD-L1 proteins have been delivered in recent years (Figure 1). Additionally, the structure of the mouse PD-1 was also disclosed. While it was assumed that PD-L1 of mouse and human origins should share a high structural similarity, experimental proofs were missing. To fill this gap, we crystallized the N-terminal PD-1-binding domain of the *m*PD-L1 protein (amino acids 19-134). The obtained crystal diffracted to the 2.5 Å resolution and contained 10 protein molecules in the asymmetric unit (Figure S1A). The crystal packing did not suggest oligomerization of the V-type domain of *m*PD-L1, consistent with its one-domain human homolog structure reported previously (Zak et al., 2015). Protein chains were arranged into two similar symmetric subunits formed by the one central PD-L1 molecule and four molecules located around the center. The subunits are contacted by the four outer PD-L1 molecules, but not the central one. Therefore, an empty core is formed in the center of the asymmetric unit (Figure S1A). The unit exhibits a pseudo-two-fold rotational symmetry around an axis parallel to the empty core.

**Figure 2:**
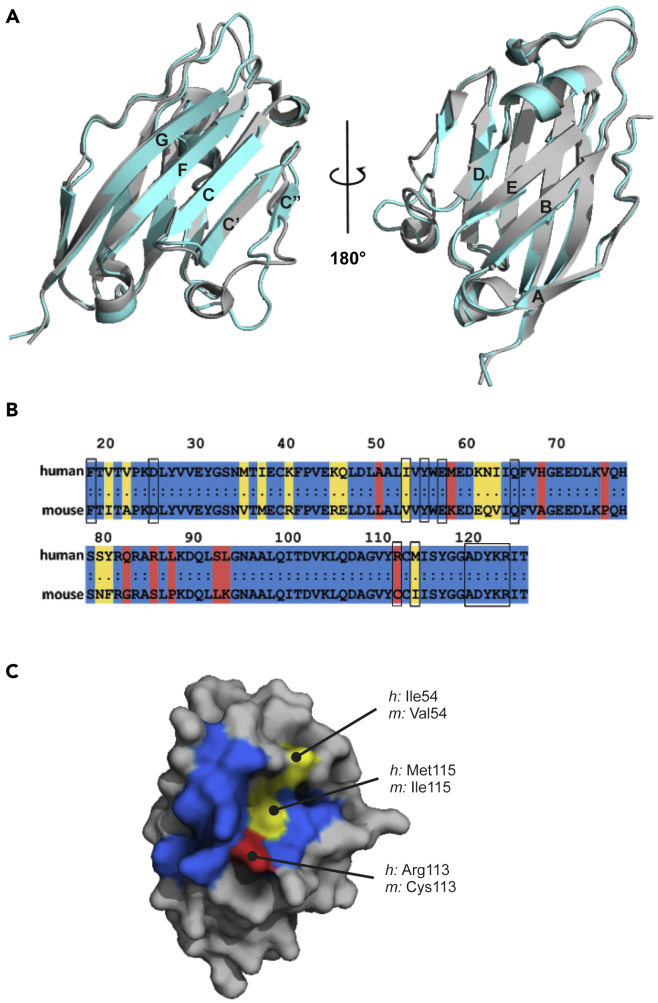
The comparison of structure and sequence of PD-1-binding domains of human and mouse PD-L1 proteins (A) The superposition of PD-1-binding domains of the human (gray, PDB: 5C3T) and mouse (cyan, PDB: 6SRU) PD-L1 proteins. Canonical Ig-strand designations ABED and GFCC’C″ are used (Zak et al., 2017). (B) Protein sequence alignment of PD-1-binding domains of human and mouse PD-L1. Conserved residues are colored blue, minor changes – yellow, significant differences – red. Black boxes indicate amino acids of *h*PD-L1 engaged in the interaction with *h*PD-1, and corresponding amino acids of *m*PD-L1. (C) Human PD-L1 structure with the surface representation of the *h*PD-L1/*m*PD-L1 sequence alignment within the interaction surface with the *h*PD-1, colored according to (B). See also Figures S1 and S2.

Similarly to the human PD-L1, the mouse protein shows the immunoglobulin-variable type topology with canonical two layers of antiparallel β-sheets (ABED and GFCC’C″, Figure 2A). Protein chains are well visible in electron density except for the two variable loops: BC (Val44-Leu47) and C”D (Leu74-Ser79). The superposition of *m*PD-L1 structures from ten copies within a single crystal unit showed only slight structural deviations in protein chains, with larger shifts in the variable loop regions (Figure S1B).

The apo-*h*PD-L1 (PDB: 5C3T) and *m*PD-L1 structures demonstrate the same general fold of the chains with root-mean-square deviation of 0.68 Å among C-alpha carbon atoms. Several significant differences within the tertiary structures are noticeable, mostly in the variable loop regions (BC and C”D loops), which are distant from the classical inhibitor-binding site and do not participate in the PD-1/PD-L1 complex formation (Figure 2A). However, the arrangement of the BC loop is debatable as a result of the flexible nature of this poorly structured region, causing many conformations or incomplete electron density for this fragment in different chains of the *m*PD-L1 structure (Figure S1B).

### Mouse PD-L1 interacts with human PD-1

The PD-1-binding domain of the human PD-L1 (Ig V-type PD-L1 domain 19-127 amino acids) shows a relatively low overall sequence identity (69.4%) and similarity (87.6%) with the mouse protein (Figure 2B). However, a detailed sequence and structure comparison within the *h*PD-1/*h*PD-L1 interaction surface demonstrates a similar arrangement of Phe19, Ala121, Asp122, Tyr123, Lys124, and Arg125 between human and mouse PD-L1 (Figures 2B and 2C). The most significant spatial rearrangement of the key amino acid within this region is a dislocation of the Tyr56 sidechain in the *m*PD-L1 structure compared to the *h*PD-L1 structure, resulting in ∼7 Å displacement of its terminal hydroxylic group (Figure S2). In addition, three amino acids involved in the formation of the *h*PD-1/*h*PD-L1 complex (Ile54, Arg113, and Met115) are changed in the *m*PD-L1 sequence to Val54, Cys113, and Ile115, respectively (Figure 2C). Among these, the Arg113/Cys113 mismatch seems the most prominent causing the inability of the salt bridge formation between *h*PD-1 and *m*PD-L1 (Zak et al., 2015). Altogether, these alterations might be sufficient to prevent the interaction of *m*PD-L1 with *h*PD-1 protein.

**Figure 3:**
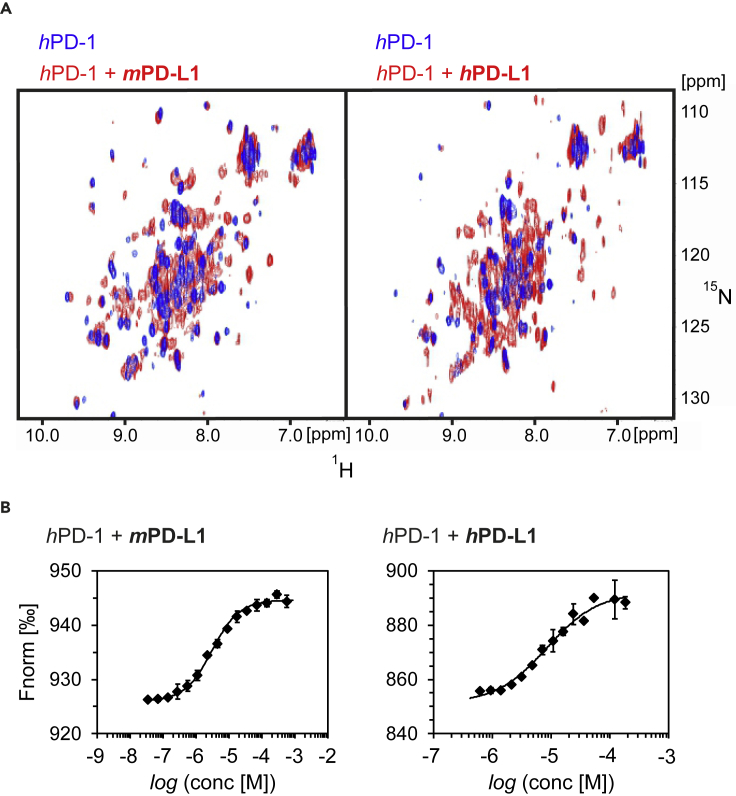
Mouse PD-L1 interacts with the human PD-1 (A) The superposition of ^1^H-^15^N HMQC spectra of the *h*PD-1 (blue) and its complex with PD-L1 (red) of either the mouse (*m*PD-L1, left panel) or human (*h*PD-L1, right panel) origin. In both 2D spectra, PD-1/PD-L1 complex formation is evidenced by the broadening of resonance signals caused by increased transverse relaxation. (B) The interaction of *h*PD-1 with either *m*PD-L1 (left) or *h*PD-L1 (right) was tested with MicroScale Thermophoresis (MST) binding assay. The resulting K_D_ value for *h*PD-1/*h*PD-L1 was 11.9 ± 2.8 μM, and 3.2 ± 0.2 μM for *h*PD-1/*m*PD-L1. All MST measurements were performed in two independent dilution series. Errors for measurement points are reported as standard deviations.

To verify the binding of *m*PD-L1 with *h*PD-1 nuclear magnetic resonance (NMR) spectroscopy was employed. For this, the 2D antagonist-nduced dissociation assay (AIDA) (Krajewski et al., 2007), used previously to assess the interaction of small molecules with *h*PD-L1 (Skalniak et al., 2017) was utilized. In the AIDA-NMR assay, the ^15^N-labeled *h*PD-1 is monitored in the ^1^H-^15^N heteronuclear multiple quantum correlation (HMQC) NMR experiment. Partial shifting and broadening of HMQC signals upon addition of the ^14^N PD-L1 indicate the formation of PD-1/PD-L1 complex, while no change implies a lack of this protein-protein interaction. In the experiment, both the human and mouse PD-L1 formed the complex with *h*PD-1 (Figure 3A). Compared to the spectrum of *h*PD-L1/*h*PD-1 complex, ^1^H-^15^N cross-peaks signals are less broadened and sharper for the *m*PD-L1/*h*PD-1 complex, which indicates a higher value of the dissociation constant of *m*PD-L1/*h*PD-1 than for *h*PD-L1/*h*PD-1 complex (Figure 3A).

The binding affinity (K_D_) between either the *m*PD-L1 or *h*PD-L1 and *h*PD-1 was further determined using MicroScale Thermophoresis (MST) assay. *h*PD-1 was covalently labeled with a fluorophore to allow for monitoring of the thermal stability of the target protein. The interaction of the labeled *h*PD-1 with its ligand, such as PD-L1, changes the stability of the protein observed as a change of the Fnorm [‰] parameter. Labeled *h*PD-1 was incubated with increasing concentrations of unlabeled *h*PD-L1 or *m*PD-L1. For both proteins a dose-dependent change in the Fnorm [‰] value was observed, indicating the interaction between them (Figure 3B). Resulting data points from two independent dilution series were fitted with K_D_ model resulting in K_D_ values of 11.9 ± 2.8 μM for the *h*PD-1/*h*PD-L1 interaction and 3.2 ± 0.2 μM for the *h*PD-1/*m*PD-L1 (Figure 3B).

### Mouse PD-L1 forms a functional immune checkpoint with human PD-1

**Figure 4:**
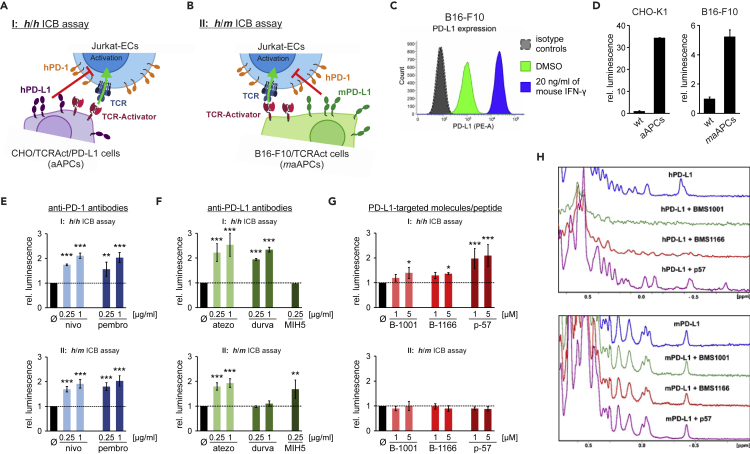
The *h/m* and *h/h* PD-1/PD-L1 checkpoint functionally impair the activation of Jurkat T cells but have distinct druggability profiles (A) A schematic representation of the immune checkpoint blockade (ICB) assay, that provides the *h*/*h PD-1/PD-L1 immune checkpoint* (*h*/*h*ICB). (B) A schematic representation of the ICB assay, that provides the *h*/*m PD-1/PD-L1 immune checkpoint*(*h*/*m*ICB). (C) Flow cytometry analysis of the expression of *m*PD-L1 on the surface of *m*aAPCs, either at the basal state (green) or following the treatment with 20 ng/mL of mouse IFN-γ (blue). (D) The activation of Jurkat T cells by the cells expressing TCR-Activator compared to parental (wt) cells. (E–G) The activity of: (E), anti-PD-1 therapeutic antibodies (nivo: nivolumab, pembro: pembrolizumab), (F), anti-PD-L1 antibodies (atezo: atezolizumab, durva: durvalumab, MIH5: control anti-mouse PD-L1 antibody), and (G) PD-L1-targeted small molecules (B-1001: BMS-1001, B-1166: BMS-1166) and the macrocyclic peptide (p-57) in *h*/*h*ICB and *h*/*m*ICB assays. The graphs show luminescence values relative to untreated control cells (black bars) and reflect the relative activation of Jurkat T cells following the depicted treatments. The results are mean ± SD values from three independent experiments. Statistical analysis was performed using ANOVA with Fisher's post-hoc test for pairwise comparison with untreated control cells: ∗, p < 0.05, ∗∗, p < 0.01, ∗∗∗, p < 0.001. (H) NMR analysis of the interaction of small molecules and the peptide with *h*PD-L1 (top) and *m*PD-L1 (bottom). Aliphatic regions of ^1^H NMR spectra of the human (top) and mouse (bottom) PD-L1, alone or in the presence of the indicated molecules are shown. The colors depict ^1^H NMR spectra of apo-PD-L1 proteins (blue), and PD-L1 proteins overtitrated with BMS-1001 (green, 1:2 molar ratio), BMS-1166 (red, 1:2 molar ratio), and the peptide-57 (purple, 1:1 molar ratio). The linewidth broadening of the NMR signals and changes in the aliphatic region are visible only in *h*PD-L1 spectra implying no interactions between *m*PD-L1 and BMS compounds or peptide-57. See also Figures S3 and S4.

Given that *m*PD-L1 interacts with the *h*PD-1 protein, we were interested in whether this interaction may provide a functional immune checkpoint, as in the case of the *h*PD-L1/*h*PD-1. For this, a new setup of the previously utilized immune checkpoint blockade (ICB, otherwise called *h*/*h*ICB) assay (Magiera-Mularz et al., 2017; Skalniak et al., 2017) was prepared, in which the CHO-based artificial antigen-presenting cells (CHO/TCRAct/PD-L1, otherwise called aAPCs), which expose the hPD-L1 protein to the Jurkat T cell-like effector cells (Jurkat-ECs) (Figure 4A), were substituted with mouse aAPCs (B16-F10/TCRAct, otherwise called *m*aAPCs), prepared by overexpressing TCR-Activator on the surface of B16-F10 cells (Figure 4B). To assure a high expression of *m*PD-L1, *m*aAPCs were pre-treated for 48 hr with 20 ng/mL of mouse IFN-γ (Figure 4C). Such a setup, herein called the *h*/*m*ICB assay, provides the TCR-dependent activation of the Jurkat-ECs and presentation of endogenous *m*PD-L1 expressed by the B16-F10 cells.

The exposition of Jurkat-ECs to either aAPCs or *m*aAPCs resulted in strong TCR-dependent activation of ECs compared to Jurkat-ECs exposed to initial CHO-K1 or B16-F10 cells, as monitored by the NFAT-mediated expression of luciferase (Figure 4D). In both setups, the addition of anti-PD-1 therapeutic antibodies, nivolumab or pembrolizumab, resulted in a significant increase in the activation of Jurkat-ECs suggesting the functional activity of both the *h*PD-1/*h*PD-L1 and the *h*PD-1/*m*PD-L1 immune checkpoints (Figure 4E).

### Mouse and human PD-L1 present different profiles of druggability with therapeutic antibodies, BMS small molecules, and peptide-57

To check the species-dependent effects of the molecules designed to block PD-L1 for therapeutic purposes, the original *h*/*h*ICB assay and the *h/m*ICB assay were performed in the presence of five molecules, belonging to three distinct classes: the monoclonal antibodies (atezolizumab and durvalumab), small molecules (**BMS-1001** and **BMS-1166**), and peptides (**peptide-57**). The dose-dependent activities of these compounds were presented in our previous studies (Magiera-Mularz et al., 2017; Skalniak et al., 2017). As expected, all the compounds increased the activation of Jurkat-ECs in the standard *h*/*h*ICB assay setup, suggesting a successful blockade of the *h*PD-1/*h*PD-L1 immune checkpoint (Figures 4F and 4G, upper panels). In the assay, both FDA-approved antibodies presented the highest potency, followed by **peptide-57**. Small molecules **BMS-1001** and **BMS-1166** presented only low activity, which is in agreement with our previous report (Skalniak et al., 2017). MIH5, which is a mouse-specific anti-PD-L1 antibody, did not interfere with the *h*PD-1/*h*PD-L1 immune checkpoint (Figure 4F, upper panel).

Interestingly, neither the peptide nor small molecules were able to provide the blockade of the *h*PD-1/*m*PD-L1 immune checkpoint (Figure 4G, lower panel). The control anti-mouse PD-L1 MIH5 antibody was able to reactivate Jurkat-ECs blocked by the *m*PD-L1 (Figure 4F, lower panel). To verify these findings, a ^1^H NMR experiment was performed with the use of either the mouse or human PD-L1. The experiment confirmed the binding of **BMS-1001**, **BMS-1166**, and **peptide-57** to the *h*PD-L1, as evidenced by the changes in the NMR spectra (Figure 4H). When the *m*PD-L1 was used, no changes in the corresponding spectra were observed, indicating no interaction of these agents with the mouse PD-L1 (Figure 4H). Similar specificity toward the *h*PD-L1 was observed in ^1^H NMR experiments for four other biphenyl-based BMS compounds, i.e. **BMS-37**, **BMS-200**, **BMS-202**, and **BMS-242**. We have shown before that these compounds bind to *h*PD-L1 (Guzik et al., 2017; Skalniak et al., 2017; Zak et al., 2016). The compounds bound to *h*PD-L1, but no binding to the *m*PD-L1 was observed (Figure S3). This was further confirmed by the MST assay, where neither **BMS-1166** nor **peptide-57** was able to interact with *m*PD-L1 (Figure S4).

Out of the tested therapeutic antibodies, only atezolizumab was able to increase the activation of Jurkat T cell-like effector cells, suppressed by the *m*PD-L1 protein (Figure 4F, lower panel). Durvalumab was inactive against the *m*PD-L1.

### Atezolizumab, but not durvalumab displays anti-cancer properties in an immunocompetent mouse model

Our cell-based model predicts the differences in binding of PD-L1-targeting molecules to the *m*PD-L1 compared to the intended molecular target, which is *h*PD-L1. To verify the reliability of this prediction, the activity of the two anti-PD-L1 therapeutic antibodies, atezolizumab and durvalumab, toward the mouse PD-L1 was further analyzed.

**Figure 5:**
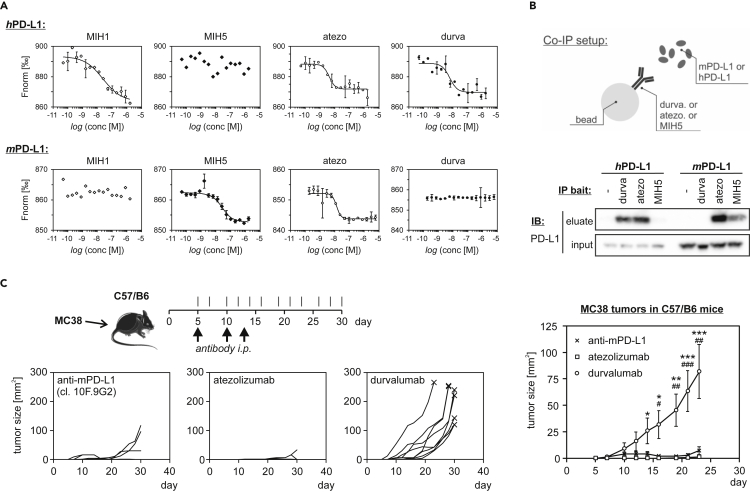
The activity of durvalumab and atezolizumab toward *h*PD-L1 and *m*PD-L1 (A) The interaction of either *h*PD-L1 (upper panels), or *m*PD-L1 (lower panels) with anti-PD-L1 antibodies was tested with MicroScale Thermophoresis (MST) binding assay. The antibodies used were: MIH1: positive control anti-*h*PD-L1, MIH5: positive control anti-*m*PD-L1, atezo: atezolizumab, durva: durvalumab. All MST measurements were performed in two independent dilution series. Errors for measurement points are reported as standard deviations. (B) Co-immunoprecipitation assay performed with the use of beads coated with the indicated anti-PD-L1 antibodies (bait), incubated with either *h*PD-L1, or *m*PD-L1 (prey). The figure shows data representative for three independent experiments. See also Figure S5. (C) MC38 tumor growth control experiment. C57/B6 mice were given 5 × 10^5^ MC38 cells s.c. and treated on days 5, 10, and 13 with anti-PD-L1 (10F.9G2; *n* = 9), atezolizumab (*n* = 9), or durvalumab (*n* = 9). Tumors were measured every 2-3 days starting on day 5. The three small plots show tumor growth data for all animals separately. The plot on the right-hand side presents mean ± SEM values from all animals within each group (*n* = 9). For statistics, ANOVA was performed with Holm post-hoc test; durva vs. 10F.9G2: #, p < 0.05, ##, p < 0.01, ###, p < 0.001; durva vs. atezo: ∗, p < 0.05, ∗∗, p < 0.01, ∗∗∗, p < 0.001.

First, binding of the antibodies to the human and mouse PD-L1 was verified using the MST assay. Fluorescently labeled *h*PD-L1 and *m*PD-L1 were incubated with increasing concentrations of antibodies. MIH1 and MIH5 antibodies were used as positive controls for *h*PD-L1 and *m*PD-L1, respectively. All of the anti-human antibodies, MIH1, atezolizumab, and durvalumab, interacted with *h*PD-L1 (Figure 5A). The determined K_D_ values were: 24.5 ± 13.6 nM for MIH1, 4.2 ± 0.9 nM for atezolizumab, and 5.9 ± 2.1 nM for durvalumab. The anti-mouse MIH5 antibody showed no binding to *h*PD-L1. In contrast, MIH1 presented no binding to the *m*PD-L1, while the binding of MIH5 was well visible (K_D_ = 13.4 ± 4.4 nM). Comparable binding affinity was observed for atezolizumab (K_D_ = 13.4 ± 2.3 nM), while durvalumab failed to interact with *m*PD-L1 (Figure 5A).

Similar species-related target specificity was determined in a co-immunoprecipitation assay, where either mouse or human PD-L1 (prey) was precipitated with beads coated with durvalumab, atezolizumab, or MIH5 as an anti-*m*PD-L1 positive control (bait). In the experiment, both durvalumab and atezolizumab showed binding to the *h*PD-L1 protein, while from this pair only atezolizumab was able to precipitate *m*PD-L1 (Figures 5B and S5). The control anti-mouse antibody MIH5 precipitated only the *m*PD-L1, but not the *h*PD-L1 (Figure 5B).

Finally, to validate the difference in activities of atezolizumab and durvalumab toward *m*PD-L1, a mouse tumor growth control experiment was performed. Immunocompetent C57/B6 mice implanted subcutaneously with the syngeneic MC38 cells were treated with atezolizumab, durvalumab, or a positive control anti-PD-L1 mouse antibody (clone 10F.9G2), and the tumor size was monitored for 25 days following the start of the treatment (Figure 5C). All animals treated with atezolizumab were clear from the tumors throughout the experiment (9/9). In contrast, almost all animals (8/9) treated with durvalumab developed the tumors that reached an average size of 80 mm^2^ on day 23 of the experiment (Figure 5C). The treatment with the *in vivo*-dedicated anti-*m*PD-L1 antibody clone 10F.9G2 also allowed for a good inhibition of the growth of MC38 tumors in C57/B6 mice, however, some animals (3/9) started to develop tumors after two weeks following the start of the treatment (Figure 5C).

### Molecular structure and amino acid sequence do not predict the druggability of the mPD-L1

To visualize the observed differences in the druggability of *h*PD-L1 and *m*PD-L1 with therapeutic antibodies, small molecules, and the peptide, the amino acid composition of PD-L1/drug interaction surfaces of *h*PD-L1 was determined based on appropriate co-crystal structures. The amino acid composition of each of the binding sites on the *h*PD-L1 was then compared to the corresponding amino acids of the *m*PD-L1 to define the residues that might underlie the observed differences in druggability.

**Figure 6:**
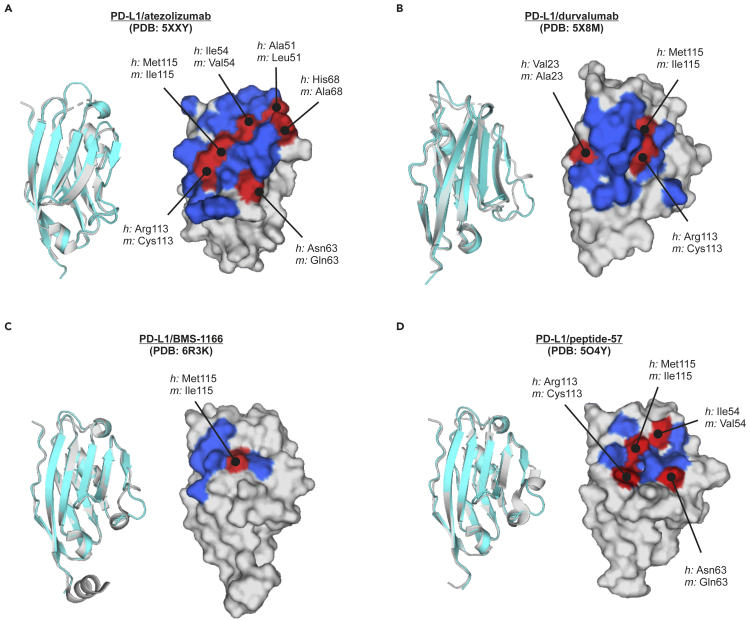
The analysis of amino acid composition of the interaction surface of PD-L1 with its blocking agents The comparison of the amino acid compositions of *h*PD-L1 and corresponding amino acids of the *m*PD-L1 at the interfaces of *h*PD-L1 with atezolizumab (A), durvalumab (B), BMS-1166 (C), and peptide-57 (D). The residues conserved between *h*PD-L1 and *m*PD-L1 are colored blue on surface representations, while the differences determined from the sequence alignment are colored red and pointed with a line. Gray surfaces represent amino acids that are apart from interaction surfaces. Structural alignments of the human (gray) and mouse (cyan) PD-L1 are shown as cartoon representations to define the orientation of the corresponding surface representations of *h*PD-L1 from given crystal structures. See also Figure S6 and Table S1.

The atezolizumab epitope within *h*PD-L1, determined from the crystal structure PDB: 5XXY, consists of 23 residues located in the CC’FG β-sheet, and loops BC, CC′, C’C″, and FG (Lee et al., 2017). Among these 23 residues, 6 are modified in the sequence of mouse PD-L1 (Figure 6A, Table S1). Durvalumab/*h*PD-L1 binding interface (from PDB: 5X8M) occupies a narrower area and is composed of 16 residues located in the strands C, F, and G and the CC′ loop, wherein 3 amino acids of the epitope are different when comparing mouse and human PD-L1 sequences (Figures 6B and Table S1). The shared epitope region for these two antibodies contains 10 residues (Tyr56, Glu58, Glu60, Asp61, Val111, Arg113, Met115, Ala121, Tyr123, and Arg125), and includes two amino acids modified between human and mouse protein (Arg113/Cys113, and Met115/Ile115).

The interaction site of **BMS-1166** and *h*PD-L1, determined from the crystal structure PDB: 6R3K consists of 8 PD-L1 residues, i.e. Ile54, Tyr56, Met115, Ala121, Asp122, Tyr123, Lys124, and Arg125 (Skalniak et al., 2017). Two out of these residues, namely Ile54 and Met115, differ between the two species, while the remaining six are the same (Figure 6C and Table S1). The binding site of **peptide-57** at the surface of *h*PD-L1 is composed of 10 amino acids, as predicted from the structure PDB: 5O4Y. Four out of these amino acids are different in the sequence of *m*PD-L1. These alterations are Ile54/Val54, Asn63/Gln63, Arg113/Cys113, and Met115/Ile115 (Figure 6D and Table S1).

### Possible molecular determinants of the species-specificity of the BMS compounds

As we have previously shown, the complexes of BMS small molecules (**BMS-8**, **BMS-37**, **BMS-200**, **BMS-202**, **BMS-1001**, and **BMS-1166**) with *h*PD-L1 are stabilized mostly by interactions of the compounds with *h*PD-L1 residues Phe19, Tyr56, Met115, Ala121, Asp122, Tyr123, Lys124, and Arg125 (Guzik et al., 2017; Skalniak et al., 2017; Zak et al., 2016). Surprisingly, these amino acids occur both in human and mouse PD-L1 sequences, except for the Met115, which is replaced by Ile115 residue in the mouse protein sequence (Figures 1B and 6C). Although the sidechains of Met115 in *h*PD-L1 and Ile115 in *m*PD-L1 overlay relatively well in the superposed structures, this replacement introduces a branched aliphatic chain to the already crowded space. Therefore, the isoleucine chain can sterically collide with the biphenyl moiety of BMS-compounds (C-C distances: 2.4 and 2.6 Å), leading to failure of the binding of the biphenyl-based inhibitors to the murine protein (Figure S6).

The second notable structural alteration is the movement of the Tyr56 sidechain, which is the largest rearrangement observed within the interaction surface between PD-L1 and **BMS-1166** when comparing the structures of apo *h*PD-L1 with apo *m*PD-L1. However, in our previous studies, we showed the Tyr56 sidechain rearrangement induced by the interaction of *h*PD-L1 with different BMS compounds. An overlay of the *m*PD-L1 and *h*PD-L1/**BMS-1166** structures shows that the rearrangement of Tyr56 necessary for binding of **BMS-1166** is prohibited in the *m*PD-L1 structure by the proximity of the Gln63 sidechain, which would also need to move out to allow for the inhibitor interaction. However, the arrangement of the strands seems to provide the space required for the reorganization of the sidechains. Therefore, the spatial arrangement of Tyr56 and Gln63 cannot be considered as the sole source for the inactivity of **BMS-1001** and **BMS-1166** toward *m*PD-L1. To check our hypothesis, we additionally tested the affinity of **BMS-37**, **BMS-202**, and **BMS-242** which are deprived of the 1,4-dioxane moiety and do not require large movement of Tyr56 upon binding. Determination of binding of **BMS-37**, **BMS-202**, and **BMS-242** to *m*PD-L1 was carried out, again, using the ^1^H NMR spectroscopy. In the resulting 1D proton spectra the linewidth broadening of the NMR lines of *m*PD-L1 was not observed, confirming a lack of interaction between these BMS compounds and *m*PD-L1.

## Discussion

The structural characterization of the PD-1/PD-L1 immune checkpoint proteins is now extensive ^19^. By now, crystal structures of the extracellular parts of the human PD-L1 containing either a single PD-1-binding domain (Zak et al., 2015) or both Ig-like domains (Chen et al., 2010; Lin et al., 2008; Zhang et al., 2017), were resolved (Figure 1). Likewise, the structures of extracellular domains of the mouse and human PD-1 proteins have been described (Cheng et al., 2013; Zhang et al., 2004). The crystal structure of the *m*PD-1/*m*PD-L2 complex was published in 2008 (Lázár-Molnár et al., 2008), likewise the mixed structure of *m*PD-1 and *h*PD-L1 (Lin et al., 2008). Next, the structure of a fully human PD-1/PD-L1 complex led to the characterization of hot-spot pockets required for the inhibition of *h*PD-1/*h*PD-L1 complex formation (Zak et al., 2015). More recently, the crystal structure of hPD-1/hPD-L2 was also published (Tang and Kim, 2019).

Out of the mouse and human PD-1/PD-L1 proteins, only the structure of the *m*PD-L1 was missing. In this paper, we fill this gap by providing the 2.5 Å-resolution crystal structure of a PD-1-binding domain of the mouse PD-L1 protein. We carried out herein a detailed structural comparison of *m*PD-L1 and *h*PD-L1 indicating their high structural similarity. We also show that human and mouse PD-L1 share enough sequence homology to allow for the interaction of the *m*PD-L1 with the *h*PD-1 protein, forming a functional immune checkpoint. This hybrid immune checkpoint was shown to functionally impair TCR-mediated activation of the reporter human Jurkat T cell-like cells, as evidenced by the observed increased activation in the presence of PD-1 blocker, nivolumab, and some other PD-1/PD-L1-blocking antibodies.

The K_D_ of the interaction between *h*PD-1 and *h*PD-L1 determined in this study (11.9 ± 2.8 μM) was in close agreement with previous data (c.a. 8 μM) (Cheng et al., 2013; Magnez et al., 2017). Despite minor sequence differences, both the *h*PD-L1 and the *m*PD-L1 are able to interact with the *h*PD-1 with comparable affinity. We, therefore, decided to check whether the *m*PD-L1 could also be targeted with the molecules designed to target the *h*PD-L1. Such an analysis is of high importance, as it might allow for choosing simple immunocompetent mouse models bearing syngeneic tumors instead of the humanized animals for pre-clinical evaluation of the bioactivity of therapeutic molecules. To our surprise, neither BMS small molecules nor the cyclic **peptide-57** were able to interact with the *m*PD-L1, while the interaction with the *h*PD-L1 was confirmed by the experimental data. This is a crucial message as it indicates that a profound *in vitro* analysis of the druggability of the *m*PD-L1 with the tested compounds is required before investing resources and time into the experiments on non-humanized animals. Moreover, the comparative analysis of the sequences and structures of mouse and human PD-L1 indicates that there is unlikely a simple rule for predicting the interaction of the *h*PD-L1-targeting ligand with the *m*PD-L1. This is well exemplified by the analysis of the two therapeutic antibodies, atezolizumab, and durvalumab, as well as BMS compounds. For both of these antibodies, amino acid sequences within their binding sites on *h*PD-L1 comprise several mismatches with the corresponding sequence of the *m*PD-L1, i.e. 3 mismatches for durvalumab, and 6 for atezolizumab, two out of which overlap for both binding sites. Despite this, it was durvalumab that did not show any interaction with the *m*PD-L1. In contrast, atezolizumab was able to interact with the *m*PD-L1, block the *m*PD-L1/*h*PD-L1 immune checkpoint in a cell-based assay, and provide inhibition of the growth of mouse tumors in a syngeneic mouse model, where the *m*PD-L1/*m*PD-1 immune checkpoint is present. Notably, the expected binding surface on *m*PD-L1 that matches the surface on *h*PD-L1 is much larger for atezolizumab (17 amino acids) than for durvalumab (13 amino acids). This may be the reason for the observed activity of atezolizumab, but not durvalumab, toward the *m*PD-L1.

These days, the interest in the examination of *in vivo* activity of emerging antibody- and non-antibody-based inhibitors of PD-1/PD-L1 is clearly increasing. Recently, several studies aiming at the evaluation of the anti-cancer properties of the biphenyl-based small molecules were presented. In a subset of these studies, syngeneic mouse models are utilized. In 2019, Zhang and co-workers published their work on **BMS-202** nanoparticles injected intravenously into BALB/c mice bearing mouse mammary gland 4T1 tumors (Zhang et al., 2019). In 2020 Hu and collaborators claimed anti-tumor effects of **BMS-202** toward B16-F10 tumors established in C57BL/6NCrl mice (Hu et al., 2020). In neither of these manuscripts was the direct binding of **BMS-202** to the *m*PD-L1 verified before moving to *in vivo* examination. In our hands, such interaction is not allowed, which somehow suggests the involvement of some off-target rather than PD-L1-dependent effects of the *in vivo* therapy presented in these studies.

In other studies, two peptide-based PD-1/PD-L1 inhibitors were tested in syngeneic mouse models (Liu et al., 2019; Sasikumar et al., 2019). In these studies, however, *in vivo* experiments were preceded by either the *in vitro* examination of *m*PD-1/*m*PD-L1 blockade (Liu et al., 2019) or the proliferation rescue and IFN-γ release assay on mouse splenocytes inhibited with the *m*PD-L1 protein (Sasikumar et al., 2019). Still, the direct binding of the tested agents to the *m*PD-L1 protein was not confirmed. Our data provided in this manuscript suggest that extreme caution is required when choosing fully mouse setups for *in vivo* evaluation of anti-cancer properties of PD-L1-targeting agents. This is because some of these agents clearly provide only human-specific therapeutic activities. Therefore, proper controls leaving no doubts as for successful targeting of the mouse PD-L1 are essential before attributing the observed biological effects to the PD-1/PD-L1 checkpoint blockade.

By now, several setups of the humanized mouse systems for immuno-oncology have been proposed (De La Rochere et al., 2018). This includes immunocompetent knock-in mice expressing either fully human or human/mouse hybrids of the proteins of interest, and immunodeficient mice that support the recapitulation of the human immune system and the engraftment of human cell lines and patient-derived xenografts (PDX). These systems are believed to provide optimal platforms for the evaluation of human PD-L1-specific drug candidates. In addition, some other setups have also been proposed, such as the implantation of the mixture of pre-activated PBMCs and human H460-Luc cells into the flank of BALB/c nude mice followed by the treatment with anti-PD-L1 peptide (Li et al., 2018). Such a setup provides a trial for eliminating the need for validating the mouse PD-L1-specificity of the experimental treatment. Still, it is not devoid of possible effects that may alter the interpretation of the results.

In conclusion, this manuscript proves for the structural similarity of the human and mouse PD-L1 protein but points out important differences in the druggability of these two proteins. It is also a guide for the followers, that a proper *in vitro* analysis done on either the isolated mouse protein or using a ‘murinized’ cell-based assay presented in this work is obligatory before starting *in vivo* studies, and that *in silico* analysis may be simply insufficient to provide reliable predictions of species specificity of the used molecule.

### Limitations of the study

In the present study, we present the first structure of the PD-1-interacting domain of a mouse PD-L1 protein. It is worth mentioning that for human PD-L1 crystal structures of the whole, two-domain extracellular part of the protein, are available (see Figure 1). As for today, the two-domain structure of the mouse PD-L1 remains unsolved. We also present that mouse PD-L1 is able to interact with the human PD-1 protein and bring an inhibitory signal toward the activated Jurkat T cells *in vitro*. Whether the functional immune checkpoint would be provided by the interaction of *m*PD-L1 with *h*PD-1 *in vivo* remains unknown.

### Resource availability

#### Lead contact

Further information and requests for resources and reagents should be directed to and will be fulfilled by the lead contact, Lukasz Skalniak (lukasz.skalniak@uj.edu.pl).

#### Materials availability

The plasmids and cell lines generated in this study will be made available on request, but we may require payment covering of the shipment fee, and/or a completed Materials Transfer Agreement if there is potential for commercial application.

#### Data and code availability

The accession number for the N-terminal PD-1-binding domain of the *m*PD-L1 protein (amino acids 19-134) reported in this paper is PDB: 6SRU.

## Methods

All methods can be found in the accompanying Transparent methods supplemental file.

## Supplemental information

Document S1. Transparent methods, Figures S1–S6, and Table S1
