# Supplementary material for: Human and mouse PD-L1: similar molecular structure, but different druggability profiles
Source: iScience. 2020 Dec 24;24(1):101960. doi: 10.1016/j.isci.2020.101960 (PMC7788105; doi:10.1016/j.isci.2020.101960)
Supplement: Document S1. Transparent methods, Figures S1–S6, and Table S1 [file mmc1.pdf]

iScience, Volume *24*

Supplemental Information

Human and mouse PD-L1: similar molecular structure, but different druggability profiles

Katarzyna Magiera-Mularz, Justyna Kocik, Bogdan Musielak, Jacek Plewka, Dominik Sala, Monika Machula, Przemyslaw Grudnik, Malgorzata Hajduk, Marcin Czepiel, Maciej Siedlar, Tad A. Holak, and Lukasz Skalniak

Supplemental data items

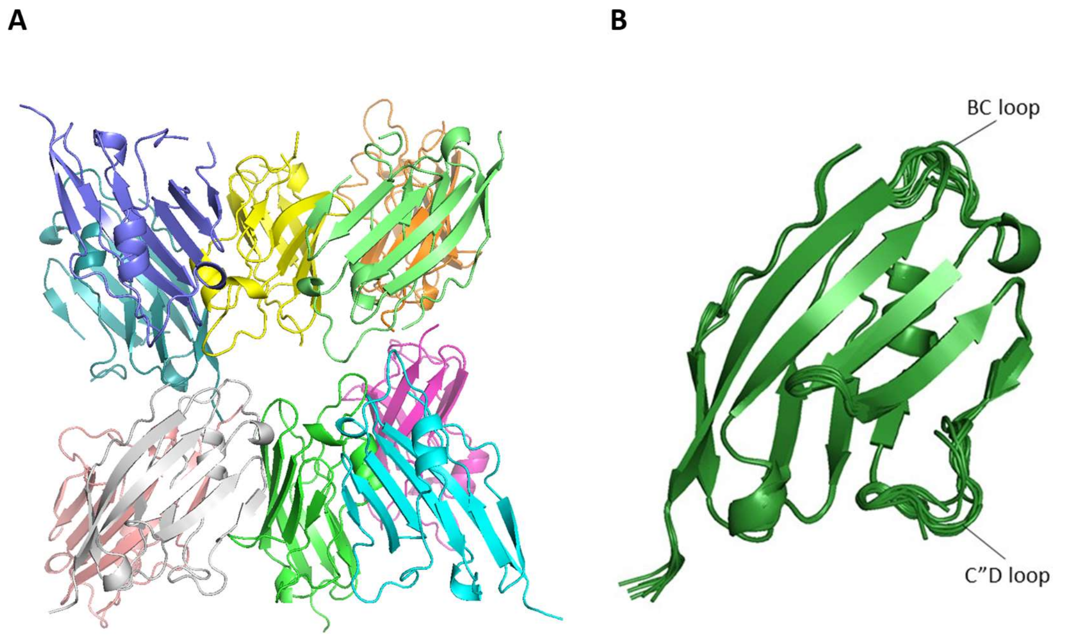

Figure S1. The overall structure of mouse apo-PD-L1. Related to Figure 2. A. The asymmetric unit is composed of 10 chains of *m*PD-L1. B. Superposition of the *m*PD-L1 chains from the ten copies. The most variable regions (BC and C”D loops) are shown.

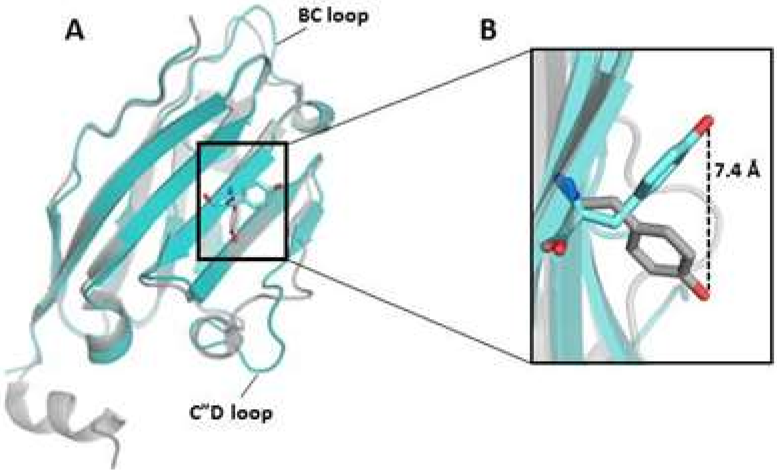

Figure S2. The rearrangement of Tyr56 sidechain between the human and mouse PD-L1 proteins. Related to Figure 2. A. The superposition of PD-1-binding domains of the human (grey, PDB: 5C3T) and mouse (cyan, PDB: 6SRU) PD-L1 proteins. B**.** Close-up view of the rearrangement of Tyr56 sidechain in the *m*PD-L1 structure compared to the *h*PD-L1 structure.

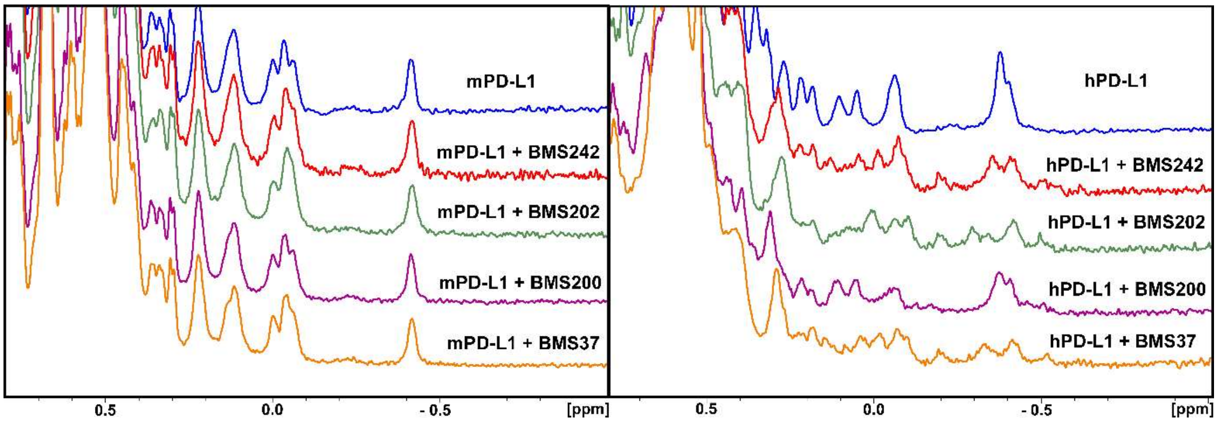

Figure S3. The aliphatic region ^{1}H NMR spectra of PD-L1 proteins. Related to Figure 4. Left panel: ^{1}H NMR spectra of apo-*m*PD-L1 (blue) and titrated *m*PD-L1 with BMS-242 (red), BMS-202 (green), BMS-200 (purple), BMS-37 (orange) at 2:1 molar ratio. Right panel: ^{1}H NMR spectra of apo-*h*PD-L1 (blue) and *h*PD-L1 with BMS-242 (red), BMS-202 (green), BMS-200 (purple), BMS-37 (orange) at 1:2 molar ratio. Linewidth broadening of the NMR signals and changes in the aliphatic region are only visible in *h*PD-L1 spectra that suggests no interaction between *m*PD-L1 and BMS compounds.

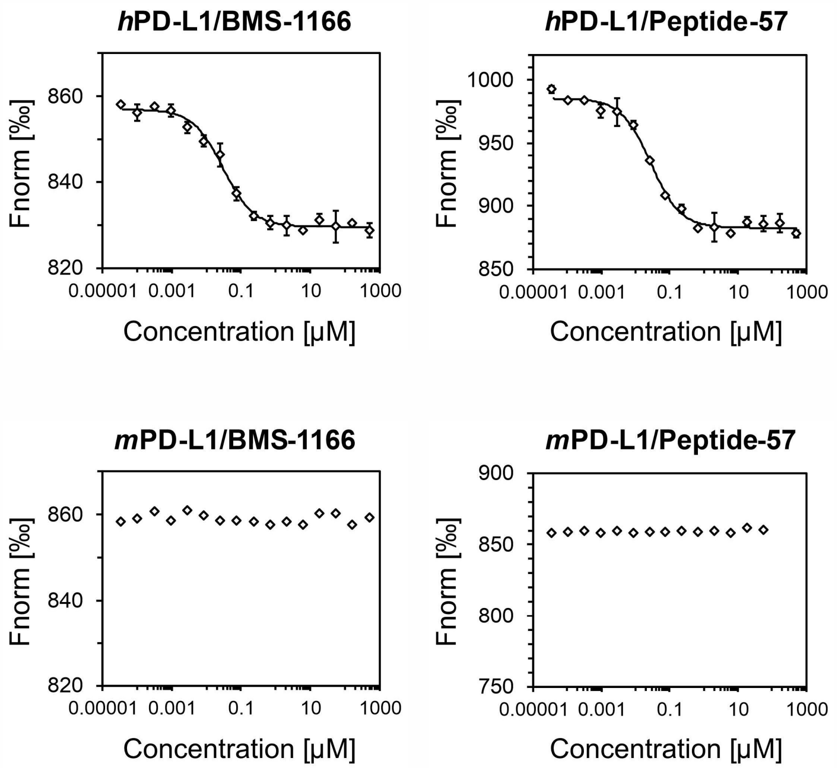

Figure S4. The interaction of BMS-1166 and peptide-57 with either *h*PD-L1 (upper panels), or *m*PD-L1 (lower panels) was tested with MicroScale Thermophoresis (MST) binding assay. Related to Figure 4.

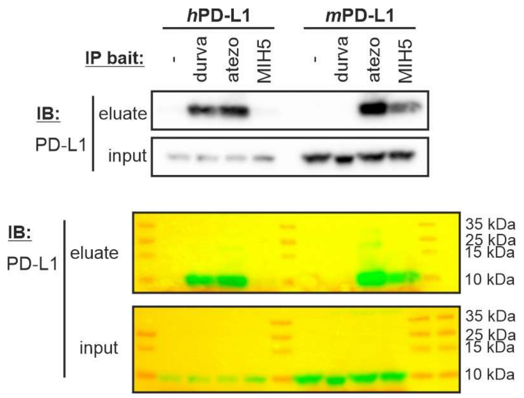

Figure S5. Co-immunoprecipitation of *h*PD-L1 and *m*PD-L1 with the use of diverse monoclonal antibodies. Related to Figure 5. Co-immunoprecipitation assay performed with the use of beads coated with the indicated anti-PD-L1 antibodies (bait), incubated with either *h*PD-L1, or *m*PD-L1 (prey). The figure shows data representative for three independent experiments. The bottom panel presents immunoblot results (green) superimposed with a bright-field image of the membrane with visible protein standards (PageRuler™ Prestained Protein Ladder, red). Molecular weights of the proteins corresponding to protein ladder bands are provided on the right side of the images.

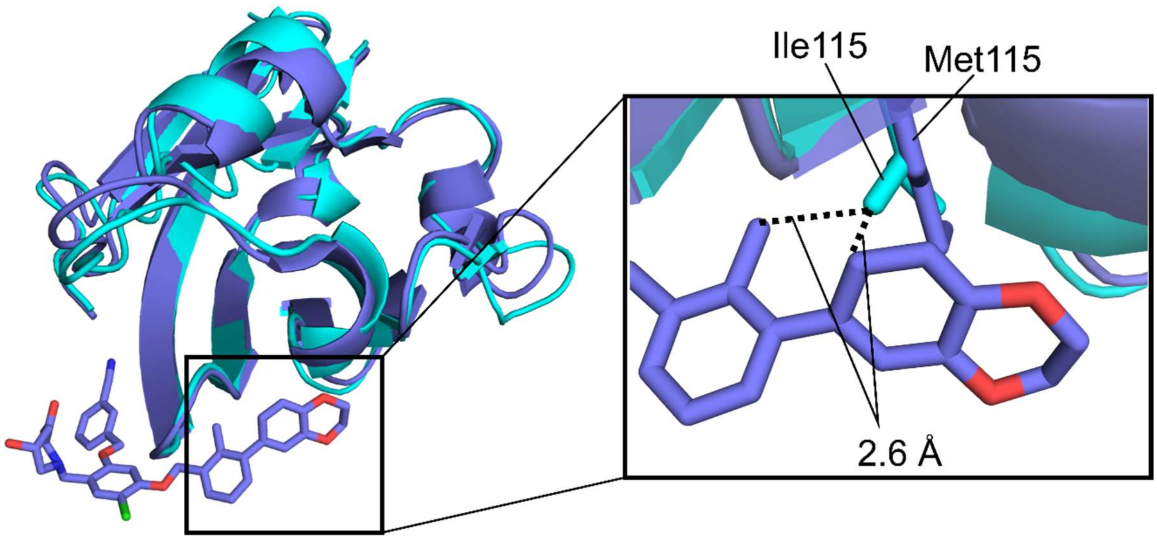

Figure S6. Overlap of *h*PD-L1/BMS-1001 (blue; PDB: 6R3K) and *m*PD-L1 (cyan; PDB: 6SRU) structures. Related to Figure 6. Ile115 (in mouse structure) can sterically collide with biphenyl moiety of BMS-compounds.

Table S1. The list of amino acids involved in the interactions between human PD-L1 (*h*PD-L1) and antibodies, small molecule BMS-1166, and peptide-57, along with the list of corresponding amino acids in the sequence of mouse PD-L1 (*m*PD-L1). Related to Figure 6. Different amino acids in mouse sequence are highlighted in bold. Adapted from Lee et al. (Lee et al., 2017)

**Atezolizumab**		**Durvalumab**	
*h*PD-L1 contact residues	analogous *m*PD-L1 residues	*h*PD-L1 contact residues	analogous *m*PD-L1 residues
A18	A18	T20	T20
E45	E45	V23	**A23**
D49	D49	D26	D26
A51	**L51**	Y56	Y56
A52	A52	E58	E58
I54	**V54**	E60	E60
Y56	Y56	D61	D61
E58	E58	V111	V111
E60	E60	R113	**C113**
D61	D61	M115	**I115**
N63	**Q63**	A121	A121
Q66	Q66	D122	D122
V68	V68	Y123	Y123
H69	**A69**	K124	K124
V111	V111	R125	R125
R113	**C113**	T127	T127
M115	**I115**		
S117	S117		
Y118	Y118		
G119	G119		
A121	A121		
Y123	Y123		
R125	R125		

**peptide-57**		**BMS-1166 $_A$PD-L1 subunit, see** (Skalniak et al., 2017)	
*h*PD-L1 contact residues	analogous *m*PD-L1 residues	*h*PD-L1 contact residues	analogous *m*PD-L1 residues
I54	**V54**	I54	**V54**
Y56	Y56	Y56	Y56
E58	E58	M115	**I115**
N63	**Q63**	A121	A121
Q66	Q66	D122	D122
V68	V68	Y123	Y123
R113	**C113**	K124	K124
M115	**I115**	R125	R125
A121	A121		
Y123	Y123		

Transparent methods

Materials

In the study, the following antibodies were used: anti-PD-1 therapeutic antibodies nivolumab (Opdivo, Bristol-Myers Squibb) and pembrolizumab (Keytruda, Merck), anti-PD-L1 therapeutic antibodies atezolizumab (Selleck Chemicals, cat. A2004) and durvalumab (Selleck Chemicals, cat. A2013), anti-human PD-L1 antibody clone MIH1 (eBioscience, cat. 14-5983-82), anti-mouse PD-L1 antibody clone MIH5 (eBioscience, cat. 14-5982-82), anti-mouse PD-L1- Purified *in vivo* PLATINUM™ Functional Grade clone 10F.9G2 (Leinco Technologies, Inc., cat. P371). The following small molecular PD-1/PD-L1-blockers developed by Bristol-Myers Squibb were used: BMS-37, BMS-200, BMS-202, BMS-242, BMS-1001, and BMS-1166, prepared as described before (Guzik et al., 2017). The macrocyclic peptide-57 was obtained from Pepmic Co., Ltd.

Protein Expression and Purification

The IgV domains of human and mouse PD-L1 protein (*h*PD-L1 residues: 18-134, C-terminal His-tag; *m*PD-L1 residues: 19-134) and the extracellular domain of human PD-1 (*h*PD-1 residues 34-150, C93S) were expressed and purified as described previously (Zak et al., 2016). Briefly, proteins were expressed in *E*. coli BL21 (DE3) strain as inclusion bodies which were collected by centrifugation, washed, and dissolved using the 6M GuHCl buffer. Proteins were refolded by drop-wise dilution into refolding buffers: 0.1 M Tris pH 8.0, 1 M L-Arg hydrochloride, 2 mM EDTA, 0.25 mM oxidized glutathione and 0.25 mM reduced glutathione for both PD-L1 proteins and 0.1 M Tris pH 8.0, 0.4 M L-Arg hydrochloride, 2 mM EDTA, 5 mM cystamine and 0.5 mM cysteamine for the *h*PD-1 protein. Refolded proteins were dialyzed 3 times over 48-72 h against buffer containing 10 mM Tris pH 8.0 and 20 mM NaCl. On the final step, proteins were concentrated and loaded to a size exclusion chromatography column HiLoad 26/600 Superdex 75 (GE Healthcare) pre-equilibrated with buffer containing 10 mM Tris pH 8.0 and 20 mM NaCl.

Crystallization of apo-mPD-L1

Purified *m*PD-L1 was concentrated to 5 mg/ml and the crystallization screening was carried out using commercially available buffer sets. Initially obtained crystals were optimized. Diffraction-quality crystals were obtained at room temperature from the condition containing: 0.2 M ammonium sulfate, 0.1 M Tris pH 7.5, 20% PEG 5000 MME. The crystal was flash-cooled in liquid nitrogen without cryoprotection.

Crystal structure determination and refinement

The X-ray diffraction data were collected at the beamline ID23-1 at the ESRF (Grenoble, France) (Nurizzo et al., 2006). The data were indexed, integrated, and scaled using XDS, XSCALE, and Aimless (Evans and Murshudov, 2013; Kabsch, 2010; Krug et al., 2012). Initial phases were obtained by molecular replacement calculated in Phaser (McCoy et al., 2007). The model building was performed in Coot and refinement was performed using Phenix or PDB-REDO server (Adams et al., 2010; Emsley and Cowtan, 2004; Joosten et al., 2014). Water molecules were added automatically and inspected manually. Coordinates and structure factors were deposited in the Protein Data Bank under accession code PDB: 6SRU.

NMR Experiments

Uniform ^{15}N labeling was obtained by expressing the proteins in the M9 minimal medium containing $^{15}NH_4Cl$ as a source of nitrogen. For NMR measurements, the buffer was exchanged to PBS pH 7.4 or 25 mM sodium phosphate pH 6.4 containing 100 mM NaCl for PD-L1 proteins or the *h*PD-1 protein, respectively. 10% (v/v) of D_2O was added to the samples to provide the lock signal. All spectra were recorded at 300 K using a Bruker Avance 600 MHz spectrometer equipped with cryo-probehead.

MST binding assay

*h*PD-1 was labeled with RED-NHS 2nd Generation dye from NanoTemper according to the attached protocol and diluted to final the concentration of 20 nM. *h*PD-1 was titrated with either *m*PD-L1 at the final concentrations from 0.0174 to 570 µM or *h*PD-L1 at the final concentration from 0.416 to 183 µM in PBS buffer with 0.005% Tween-20 and left for 1h incubation at room temperature. The MST

measurement was performed on Monolith NT.115 series using high precision capillaries at Excitation Power and MST Power of 40% in triplicates. The results were fitted with Hill's model using MO. Affinity Analysis v2.3 with a cold region at -1 s and hot region from 0.5 to 1.5 s for mPD-L1 and 4 to 5 s for *h*PD-L1.

Cell lines

Mouse skin melanoma B16-F10 cell line was obtained from the American Type Culture Collection (ATCC). Chinese hamster ovary CHO-K1 cell line was purchased from the European Collection of Authenticated Cell Cultures (ECACC). Artificial antigen-presenting (aAPCs) CHO-K1 cells expressing TCR Activator, CHO-K1 cells expressing TCR Activator, and overexpressing PD-L1 (aAPCs/PD-L1), as well as Jurkat T Effector Cells (ECs) overexpressing PD-1 and containing the Luciferase gene controlled by NFAT Response Element (NFAT-RE) were acquired from Promega. All cell lines were cultured in RPMI-1640 (Biowest) supplemented with 10% Fetal Bovine Serum (FBS, Biowest) and 200 mM L-Glutamine (Biowest). To sustain the stable transfection, aAPCs were cultured in the presence of Hygromycin B Gold (50 µg/ml, InvivoGen), while aAPCs/PD-L1 and ECs were cultured in the presence of Hygromycin B Gold (50 µg/ml, InvivoGen) and G418 (250 µg/ml, InvivoGen). The overexpression of PD-1 and PD-L1 was confirmed by flow cytometry, while the expression of TCR Activator was confirmed by western blotting. MC38 cells (Kerafast) were cultured in DMEM containing 4.5 g/l of glucose, and supplemented with 10% FBS and 200 mM L-Glutamine. The cells were routinely controlled for *Mycoplasma sp.* contamination with the PCR-based method (van Kuppeveld et al., 1992).

*The preparation of the B16-F10/TCRAct cells (*m*aAPCs)*

For the generation of the B16-F10/TCRAct cells (*ma*APCs), B16-F10 cells were transfected with the pcDNA3.1 (+) plasmid (Invitrogen) encoding TCRAct hybrid protein, composed of the human CD5 membrane-localization sequence, scFv fragment of the OKT3 antibody, and the transmembrane fragment of the human CD28 (CD5-scFv OKT3-CD28) (Leitner et al., 2010). The transfection was done using Lipofectamine 2000 (Invitrogen). Stable transfectants were selected with 1 mg/ml of G418. The expression of TCRAct was verified first by the functional assay (the *h*/*m*ICB assay, see below) and by the western blot with the use of anti-CD28 antibody clone EPR22076. For the experiments, the clone named 3C4 was used.

Flow cytometry

For the flow cytometry analysis the cells were detached from the plates with TrypLE Select Enzyme (Gibco), placed on ice, washed two times with Flow Cytometry Staining Buffer (eBioscience) and stained with primary fluorophore-conjugated antibodies: PE-conjugated rat anti-mouse PD-L1 antibody clone MIH5 (eBioscience, cat. 12-5982-82), PE-conjugated rat IgG2a kappa isotype control clone eBR2a (eBioscience, cat. 12-4321-80), APC-conjugated mouse anti-human PD-L1 antibody clone MIH1 (eBioscience, cat. 17-5983-42), APC-conjugated mouse IgG1 kappa isotype control clone P3.6.2.8.1 (eBioscience, cat. 17-4714-82). Samples were analyzed with FACSVerse flow cytometer (Becton Dickinson, BD) and BD FACSuite™ Software.

Western blotting

The cell lysates were prepared with RIPA buffer (Sigma-Aldrich) containing Protease Inhibitor Cocktail (Sigma-Aldrich). The lysates were separated by 12% SDS-PAGE (TGX™ FastCast™ Acrylamide Kit, Bio-Rad) and transferred into a PVDF membrane (Merck Millipore) using a Mini Trans-Blot® Cell system (Bio-Rad) at 100 V for 1.5 h on ice. The membranes were incubated in 4% (m/v) bovine serum albumin (BioShop) in TBS buffer containing 0.1% (v/v) Nonidet P-40 (BioShop) at RT for 1 h. Then, the membranes were incubated with specific primary antibody at 4°C overnight. Following three washes in TBS-N, the secondary horseradish peroxidase (HRP)-conjugated antibody was applied for 1 h at RT. After three additional washes in TBS-N, the visualization of protein with Clarity Western ECL Substrate (Bio-Rad) and ChemiDoc MP Imaging System (Bio-Rad) was carried out according to the manufacturer's instructions.

The proteins of interest were detected with primary antibodies: rabbit monoclonal anti-CD28 antibody at a 1:1,000 dilution (EPR22076, Abcam, cat. ab243228), rabbit monoclonal anti-α-tubulin at 1:2,000 (11H10, Cell Signaling Technology, CST, cat. 2125), and the goat HRP-linked anti-rabbit secondary antibody at 1:3,000 (CST, cat.7074).

Immune Chceckpoint Blockade (ICB) assay

Artificial antigen presenting cells: CHO/TCRAct/PD-L1(aAPCs, used in the *h*/*h*ICB assay) and B16-F10/TCRAct (*ma*APCs, used in the *h*/*m*ICB assay), as well as the initial CHO K1 and B16-F10 cells were either seeded on 96-well plates at the density of 10,000 cells/well and processed, or seeded at the density of 2,000 cells/well and treated with 20 ng/ml of mouse recombinant IFN-γ (Thermo Fisher Scientific) for 48 hours before processing. The culture medium was discarded and the wells were gently washed two times using fresh portion of culture medium to remove residual IFN-γ. Jurkat T cells overexpressing PD-1 and luciferase gene controlled by the NFAT-response element (Jurkat Effector Cells, Jurkat-ECs) were subsequently added to the aAPCs and *ma*APCs at the density 20,000 cells/well, and cells were incubated in the assay buffer (99% RPMI 1640, 1% FBS) in the presence of PD-1/PD-L1 inhibitors for 24h. The luminescence was measured after the addition of Bio-Glo™ Assay reagent (Promega), according to the manufacturer's instructions, using the Infinite M200 plate reader (TECAN).

The stock solutions of small-molecules and the peptide-57 were first prepared in DMSO (2 mM and 10 mM stock solutions) and subsequently diluted in the assay buffer at a ratio 1:1,000. Due to the addition of the ECs, the final concentrations of the compounds were 1 μM and 5 μM, as indicated in the figures. The dilutions of the monoclonal antibodies were performed in the assay buffer prior to the experiments, to obtain the final concentrations of 0.25 μg/ml and 1 μg/ml.

Co-immunoprecipitation

For immunoprecipitation 25 μg/ml of antibodies (durvalumab/atezolizumab/MIH5) in PBS with 0,02% (v/v) Tween 20 (Bio-Rad) were incubated for 10 minutes with Dynabeads™ Protein G (Thermo Fisher Scientific, cat. 10004D). Then, Protein G-Abs complexes were incubated with IgV domains of *m*PD-L1 or *h*PD-L1 (see Protein Expression and Purification section) in the same concentrations of 2μg/ml in PBS with 1% (m/v) bovine serum albumin (BioShop) for 15 minutes. Samples were washed three times with 200 μl PBS and suspended in 100 μl of PBS. To get rid of beads coated with Protein G, the samples were denaturated with SDS-PAGE sample buffer and analyzed on WB with PD-L1 (N) Rabbit Polyclonal Primary Antibody at a 1:2,000 dilution (Abiocode, cat. R0586-1) and HRP-Linked Anti-Rabbit Secondary Antibody at a 1:3,000 dilution (CST, cat. 7074).

Syngeneic mouse model

For the *in vivo* study, 6–10-weeks-old C57BL/6 mice (Janvier Labs) were used. Tumor cell line MC38 cells ($5x10^5$ cells) were injected subcutaneously in 50 μl of sterile PBS into a single flank of each experimental mouse. At the day 5 following the injection, three mice that developed measurable tumors were excluded from the experiment. The mice were randomly divided into three groups, each containing 9 animals. Therapeutic antibodies, atezolizumab and durvalumab, as well as control anti-mouse PD-L1 antibody clone 10F.9G2 were injected intraperitonealy at the dose of 200 μg in 200 μl of physiological salt at the days 5, 9, and 13 following the injection. The growth of the tumors was monitored every 2-3 days by measuring two perpendicular diameters of the tumors and calculating tumor size as the tumor surface equal to the product of these diameters (mm^2). The mice were sacrified either when the tumor exceeded the size of 250 mm^2 or at the day 30 of the experiment. The tumor growth data was analyzed using the TumGrowth online tool (Enot et al., 2018) with the following settings: no Automatic outlier detection, no Check group-heteroskedacity. Statistical significance was tested using ANOVA and Holm post-hoc test for pairwise comparisons.

Supplemental References

Adams, P.D., Afonine, P. V, Bunkóczi, G., Chen, V.B., Davis, I.W., Echols, N., Headd, J.J., Hung, L.-W., Kapral, G.J., Grosse-Kunstleve, R.W., McCoy, A.J., Moriarty, N.W., Oeffner, R., Read, R.J., Richardson, D.C., Richardson, J.S., Terwilliger, T.C., Zwart, P.H., 2010. PHENIX: a comprehensive Python-based system for macromolecular structure solution. Acta Crystallogr. D. Biol. Crystallogr. 66, 213–21.

Emsley, P., Cowtan, K., 2004. Coot: model-building tools for molecular graphics. Acta Crystallogr. D. Biol. Crystallogr. 60, 2126–32.

Enot, D.P., Vacchelli, E., Jacquelot, N., Zitvogel, L., Kroemer, G., 2018. TumGrowth: An open-access web tool for the statistical analysis of tumor growth curves. Oncoimmunology 7, e1462431.

Evans, P.R., Murshudov, G.N., 2013. How good are my data and what is the resolution? Acta Crystallogr. D. Biol. Crystallogr. 69, 1204–14.

Joosten, R.P., Long, F., Murshudov, G.N., Perrakis, A., 2014. The PDB_REDO server for macromolecular structure model optimization. IUCrJ 1, 213–20.

Kabsch, W., 2010. XDS. Acta Crystallogr. D. Biol. Crystallogr. 66, 125–32.

Krug, M., Weiss, M.S., Heinemann, U., Mueller, U., 2012. XDSAPP : a graphical user interface for the convenient processing of diffraction data using XDS. J. Appl. Crystallogr. 45, 568–572.

Leitner, J., Kuschei, W., Grabmeier-Pfistershammer, K., Woitek, R., Kriehuber, E., Majdic, O., Zlabinger, G., Pickl, W.F., Steinberger, P., 2010. T cell stimulator cells, an efficient and versatile cellular system to assess the role of costimulatory ligands in the activation of human T cells. J. Immunol. Methods 362, 131–41.

McCoy, A.J., Grosse-Kunstleve, R.W., Adams, P.D., Winn, M.D., Storoni, L.C., Read, R.J., 2007. Phaser crystallographic software. J. Appl. Crystallogr. 40, 658–674.

Nurizzo, D., Mairs, T., Guijarro, M., Rey, V., Meyer, J., Fajardo, P., Chavanne, J., Biasci, J.C., McSweeney, S., Mitchell, E., 2006. The ID23-1 structural biology beamline at the ESRF. J. Synchrotron Radiat. 13, 227–38.

van Kuppeveld, F.J., van der Logt, J.T., Angulo, A.F., van Zoest, M.J., Quint, W.G., Niesters, H.G., Galama, J.M., Melchers, W.J., 1992. Genus- and species-specific identification of mycoplasmas by 16S rRNA amplification. Appl. Environ. Microbiol. 58, 2606–2615.
